# Novel Snakebite Therapeutics Must Be Tested in Appropriate Rescue Models to Robustly Assess Their Preclinical Efficacy

**DOI:** 10.3390/toxins12090528

**Published:** 2020-08-19

**Authors:** Cecilie Knudsen, Nicholas R. Casewell, Bruno Lomonte, José María Gutiérrez, Sakthivel Vaiyapuri, Andreas H. Laustsen

**Affiliations:** 1 Department of Biotechnology and Biomedicine, Technical University of Denmark, 2800 Kongens Lyngby, Denmark; 2 BioPorto Diagnostics, 2900 Hellerup, Denmark; 3 Centre for Snakebite Research & Interventions, Liverpool School of Tropical Medicine, Liverpool L3 5QA, UK; nicholas.casewell@lstmed.ac.uk; 4 Instituto Clodomiro Picado, Facultad de Microbiologia, Universidad de Costa Rica, 11501 San José, Costa Rica; bruno.lomonte@ucr.ac.cr (B.L.); jose.gutierrez@ucr.ac.cr (J.M.G.); 5 School of Pharmacy, University of Reading, Reading RG6 6UB, UK; s.vaiyapuri@reading.ac.uk

**Keywords:** Snakebite envenoming, rescue assays, preincubation assays, pre-clinical evaluation, toxicokinetics, pharmacokinetics, envenoming therapy

## Abstract

In the field of antivenom research, development, and manufacture, it is often advised to follow the World Health Organization’s (WHO) guidelines for the production, control, and regulation of snake antivenom immunoglobulins, which recommend the use of preincubation assays to assess the efficacy of snakebite therapeutics. In these assays, venom and antivenom are mixed and incubated prior to in vivo administration to rodents, which allows for a standardizable comparison of antivenoms with similar characteristics. However, these assays are not necessarily sufficient for therapeutics with significantly different pharmacological properties than antibody-based antivenoms, such as small molecule inhibitors, nanoparticles, and other modalities. To ensure that the in vivo therapeutic utility of completely novel toxin-neutralizing molecules with no history of use in envenoming therapy and variable pharmacokinetics is properly evaluated, such molecules must also be tested in preclinical rescue assays, where rodents are first challenged with appropriate doses of venoms or toxins, followed by the administration of neutralizing modalities after an appropriate time delay to better mimic the real-life scenarios faced by human snakebite victims. Such an approach takes the venom (or toxin) toxicokinetics, the drug pharmacokinetics, and the drug pharmacodynamics into consideration. If new modalities are only assessed in preincubation assays and not subjected to evaluation in rescue assays, the publication of neutralization data may unintentionally misrepresent the actual therapeutic efficacy and suitability of the modality being tested, and thus potentially misguide strategic decision making in the research and development of novel therapies for snakebite envenoming.

Snakebite envenoming has gained renewed attention after it was recently reinstated on the World Health Organization’s (WHO’s) List of Neglected Tropical Diseases [1], and the development of novel therapeutics for envenoming has fortunately been stated as an important strategic goal for reducing the global burden of this debilitating affliction [2]. Researchers worldwide may now therefore be increasingly incentivized to pursue the exploration of novel concepts and molecules for their therapeutic utility in treating snakebite envenoming. While it is encouraging that scientific efforts are strengthening novel research on therapeutics for envenoming and that a range of fundamentally different strategies are being investigated [3,4], the proper preclinical evaluation of these novel therapies must be carefully considered.

The reported efficacy of new therapeutics must be accompanied by a careful examination of the assumptions underlying the assays used for evaluation. As a first step in the assessment of the preclinical efficacy of a drug for snakebite envenoming, preincubation assays must be performed (Figure 1) according to WHO guidelines [5]. These are excellent for gauging the feasibility of a drug or molecule being effective in vivo, and candidate venom inhibitors that fail in preincubation assays are likely not worth testing further [6,7]. From a pharmacological point of view, preincubation assays are also the most reproducible way of determining the median effective dose (ED_50_) of a venom inhibitory drug. However, this is clearly not enough for a thorough preclinical evaluation, because the pharmacokinetic and pharmacodynamic profiles of these inhibitors, vis-à-vis the toxicokinetic profile of venoms or toxins, should also be taken into consideration. The corollary is that it is especially important for novel treatment modalities that have shown promising results in vitro to be tested in vivo, not only in preincubation assays but also in rescue assays, i.e., those in which venom is injected first and the inhibitory drug is administered after a time-lapse (see Figure 1). Indeed, an argument could be made that existing, conventional, immunoglobulin-based antivenoms would also benefit from re-evaluation in such assays given the complexities associated with the determination of what extent the varying preclinical efficacies reported in preincubation assays reflect efficacy in the more realistic scenario of rescue assays [8].

Rescue assays more accurately reflect real-life envenoming and elucidate the influence of venom (or toxin) toxicokinetics, drug pharmacodynamics, and drug pharmacokinetics. For example, if a drug is rapidly eliminated from circulation, it will be unable to bind and inhibit venom toxins, hence requiring a modification of the drug to extend its half-life. This would be evident in a rescue experiment, but it would not be evident in a preincubation assay. Moreover, new therapeutics for snakebite envenoming may include drugs that do not directly bind and inhibit venom toxins; instead, they could modify endogenous physiological processes, cellular receptors, and/or intracellular signaling molecules in the body, thus combating the toxicity of venom through different mechanisms. For these types of drug, the preincubation assay is not useful, whereas rescue experiments provide a more realistic scenario for assessing their therapeutic potential. Furthermore, rescue experiments allow for venom and drugs to be administered to an animal by different routes, e.g., venom by a subcutaneous route and a drug by the intravenous, subcutaneous, or oral routes, depending on its properties (see, for example, [9]). This will likely better reproduce the circumstances of a snakebite and the ensuing therapeutic intervention, though further research is undoubtedly required to more accurately model the toxicokinetic properties of murine envenoming to mimic those of a human snakebite. Ultimately, both preincubation assays and rescue assays are valuable models of envenoming, and they should be viewed as complementary, with new treatment modalities being evaluated with both types of assay and the need to follow the principle of the 3Rs (replacement, reduction, and refinement) in animal experimentation always kept in mind [10]. When interpreting the results of such assays, it should always be considered that animal models do not necessarily reflect the dynamics of envenoming in humans. In the long term, the preclinical evaluation of novel therapeutics should be followed by appropriate clinical testing.

As the battle against snakebite envenoming gains momentum and becomes further integrated into the global health agenda, it is more important than ever that decision-makers have the most accurate information at their disposal concerning new therapeutic approaches for tackling envenoming. For this reason, the experimental approaches used to evaluate the preclinical efficacy of new snakebite therapies should be carefully considered, and a high level of transparency regarding the limitations of studies assessing novel treatment modalities must be prioritized. In this way, the limited resources available for the field can be dedicated to those therapeutic candidates that offer the greatest chance of delivering effective, safe, and affordable treatments to save the lives and limbs of the world’s impoverished snakebite victims.

## Figures and Tables

**Figure 1:**
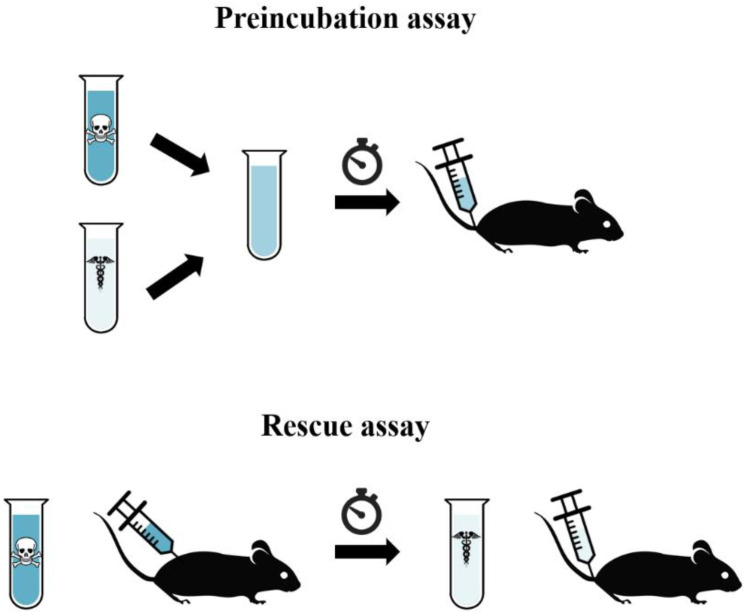
Schematic representation of preincubation and rescue assays. In preincubation assays, venom(s) or toxin(s) are mixed with antitoxins or inhibitors, and they are preincubated prior to administration in a rodent. In rescue assays, the rodent is first injected with venom(s) or toxins. After a range of delayed time points, antitoxins or inhibitors are then administered via a route appropriate for the drug being tested.

## References

[1] Chippaux J.-P. (2017). Snakebite envenomation turns again into a neglected tropical disease!. J. Venom. Anim. Toxins Trop. Dis..

[2] Williams D.J., Faiz M.A., Abela-Ridder B., Ainsworth S., Bulfone T.C., Nickerson A.D., Habib A.G., Junghanss T., Fan H.W., Turner M. (2019). Strategy for a globally coordinated response to a priority neglected tropical disease: Snakebite envenoming. PLoS Negl. Trop. Dis..

[3] Knudsen C., Laustsen A.H. (2018). Recent Advances in Next Generation Snakebite Antivenoms. Trop. Med. Infect. Dis..

[4] Bulfone T.C., Samuel S.P., Bickler P.E., Lewin M.R. (2018). Developing Small Molecule Therapeutics for the Initial and Adjunctive Treatment of Snakebite. J. Trop. Med..

[5] World Health Organization (2018). WHO Guidelines for the Production, Control and Regulation of Snake Antivenom Immunoglobulins.

[6] Ainsworth S., Menzies S., Casewell N.R., Harrison R.A. (2020). An analysis of preclinical efficacy testing of antivenoms for sub-Saharan Africa: Inadequate independent scrutiny and poor-quality reporting are barriers to improving snakebite treatment and management. PLoS Negl. Trop. Dis..

[7] Richard G., Meyers A.J., McLean M.D., Arbabi-Ghahroudi M., MacKenzie R., Hall J.C. (2013). In Vivo Neutralization of α-Cobratoxin with High-Affinity Llama Single-Domain Antibodies (VH Hs) and a VH H-Fc Antibody. PLoS ONE.

[8] Laustsen A.H., Karatt-Vellatt A., Masters E.W., Arias A.S., Pus U., Knudsen C., Oscoz S., Slavny P., Griffiths D.T., Luther A.M. (2018). In vivo neutralization of dendrotoxin-mediated neurotoxicity of black mamba venom by oligoclonal human IgG antibodies. Nat. Commun..

[9] Gutiérrez J.M., Lewin M.R., Williams D.J., Lomonte B. (2020). Varespladib (LY315920) and Methyl Varespladib (LY333013) Abrogate or Delay Lethality Induced by Presynaptically Acting Neurotoxic Snake Venoms. Toxins.

[10] Gutiérrez J.M., Solano G., Pla D., Herrera M., Segura Á., Vargas M., Villalta M., Sánchez A., Sanz L., Lomonte B. (2017). Preclinical Evaluation of the Efficacy of Antivenoms for Snakebite Envenoming: State-of-the-Art and Challenges Ahead. Toxins.

